# The potency of luliconazole against clinical and environmental *Aspergillus nigri* complex

**Published:** 2019-12

**Authors:** Sahar Hivary, Mahnaz Fatahinia, Marzieh Halvaeezadeh, Ali Zarei Mahmoudabadi

**Affiliations:** 1 Department of Medical Mycology, School of Medicine, Ahvaz Jundishapur University of Medical Sciences, Ahvaz, Iran; 2 Infectious and Tropical Diseases Research Center, Health Research Institute, Ahvaz Jundishapur University of Medical Sciences, Ahvaz, Iran

**Keywords:** Black *Aspergillus* strains, Luliconazole, Clinical and environmental isolates, Antifungal profile

## Abstract

**Background and Objectives:**

Black *Aspergillus* strains including, *Aspergillus niger* and *A. tubingensis*, are the most cause of otomycosis with worldwide distribution. Although, amphotericin B was a Gold standard for the treatment of invasive fungal infection for several decades, it gradually replaced by fluconazole and /or voriconazole. Moreover, luliconazole, appears to offer the best potential for *in vitro* activity against black *Aspergillus* strains. The aim of the present study was to compare the *in vitro* activity luliconazole, with commonly used antifungals against clinical and environmental strains of black *Aspergillus*.

**Materials and Methods:**

Sixty seven (37 clinical and 30 environmental) strains of black *Aspergillus* were identified using morphological and molecular technique (β-Tubulin gene). In addition, antifungal susceptibility test was applied according to CLSI M38 A2. The results were reported as minimum inhibitory concentration (MIC) or minimum effective concentration (MEC) range, MIC_50_ or MEC_50_, MIC_90_ or MEC_90_ and MIC geometric (GM) or MECGM.

**Results:**

*Aspergillus niger* was the common isolate followed by, *A. tubingensis* in both clinical and environmental strains. The lowest MIC range, MIC_50_, MIC_90_, and MICGM was attributed to luliconazole in clinical strains. The highest resistant rate was found in amphotericin B for both clinical (86.5%) and environmental (96.7%) strains whereas 54.1% of clinical and 30% of environmental isolates were resistant to caspofungin. Clinical strains of *Aspergillus* were more sensitive to voriconazole (86.7%) than environmental strains (70.3%). On the other hand, 83.8% of clinical and 70% of environmental isolates were resistant to posaconazole.

**Conclusion:**

Luliconazole versus amphotericin B, voriconazole, posaconazole and caspofungin is a potent antifungal for *Aspergillus* Nigri complex. The *in vitro* extremely antifungal efficacy against black *Aspergillus* strains of luliconazole, is different from those of other used antifungals.

## INTRODUCTION

Luliconazole (Luzu®), (-)-(E)-[(4R)-4-(2,4-dichlorophe-nyl)-1,3-dithiolan-2-ylidene] (1H-imidazol-1-yl) acetonitrile), is an imidazole antifungal with molecular formula: C_14_H_9_Cl_2_N_3_S_2_ (1). Luliconazole was basically introduced as anti-dermatophytic antifungal in Japan and India (1, 2). However, it has demonstrated activity *in vitro* against multiple *Aspergillus* species, including *Aspergillus fumigatus* (3, 4), *A. terreus* (4, 5), *A. flavus* (4, 6), *A. niger* (4) and *A. tubingensis* (4). The availability of a novel antifungal, luliconazole, appears to offer the potential for improved therapy for a wide range of invasive fungal infections, including aspergillosis, dermatophytosis, and onychomycosis (2, 7, 8).

While, amphotericin B was a Gold standard in the first-line treatment of invasive fungal infections for several decades (9), it has been replaced by several new antifungals including, voriconazole, posaconazole and caspofungin (10, 11). Voriconazole was presented as the primary therapy for invasive pulmonary aspergillosis in a clinical trials (12). Further studies have shown that posaconazole is a useful antifungal for invasive fungal infection including aspergillosis (13). On the other hand, during 2–3 last decades, caspofungin was developed to improve the prognosis of invasive aspergillosis (14).

The section Nigri (*A. niger*, sensu lato) contains more than 19 accepted species including, *A. niger, A. tubingensis, A. awamory, A. welwitschiae, A. acidus, A. brasiliensis* and others (15–18). The *Aspergillus* strains in this section are comprised of several closely related species, and their identification based on sequence analyses of β-tubulin gene (4). *Aspergillus niger* and *A. tubingensis* strains frequently isolated from clinical infections (16, 19–21). Black *Aspergillus* strains cause several types of aspergillosis among predisposed patients (22–25). Out of them, otomycosis is the most common cutaneous infection caused by black *Aspergillus* strains (4, 20).

The increasing of fungal opportunistic infections among patients receiving intensive chemotherapy, hematological malignancies and transplant patients was remarkable during last decades (10, 23, 26–28). Invasive *Aspergillus* infections are one of the life threatening human disease. On the other hand, some species of *Aspergillus* have inherent resistance to some antifungal agents (29). Moreover, some species have raised minimum inhibitory concentration (MIC) against specific antifungals. As a results, infection prevention consultant and the best choice antifungal are common clinical challenges.

The aim of the present study was to compare the *in vitro* activity of a novel antifungal agent, luliconazole, with amphotericin B, voriconazole, posaconazole and caspofungin against clinical and environmental strains of black *Aspergillus*. Furthermore, the potency of each antifungal against clinical and environmental isolates was compared.

## MATERIALS AND METHODS

### Fungal isolates.

Thirty seven clinical isolates of black *Aspergillus* strains were previously isolated from otomycosis samples, identified based on morphology characteristics and preserved at Medical Mycology laboratory affiliated to Ahvaz Jundishapur University of Medical Sciences. This project was approved by the ethical committee of Ahvaz Jundishapur University of Medical Sciences (IR.AJUMS. REC.1396.1066).

Environmental strains of black *Aspergillus* (30 strains) were trapped from airborne spores using Sabouraud dextrose agar (SDA) (BioLife, Italia) plates. Primary screening of black *Aspergillus* strains was applied based on macroscopic (Black colony) and microscopic morphology. All strains (clinical and environmental) were subcultured on SDA and re-identified using molecular tests.

### DNA extraction.

All strains (clinical and environment isolates) were subcultured on SDA plates and incubated at 29ºC for 24–48 hours. Mycelia were collected in cryo-tubes containing 300 μL lysis buffer and 0.46 g glass beads and kept at 4ºC for 72 hours. The tube contents were homogenized using a Speed-Mill PLUS Homogenizer (Analytikjena, Germany) for 6 minutes (3 cycles) and boiled at 100ºC for 20 minutes. 300 μL of sodium acetate (3M) was added to each tube and stored at −20ºC for 10 minutes. Supernatants were removed after a centrifugation at 12000 rpm for 10 minutes. DNA was purified using phenol-chloroform-isoamyl alcohol (Merck, Germany) according to a protocol devised by Makimura et al. (30). Finally purified DNA was preserved at −20ºC for further tests.

### Molecular identification.

β-Tubulin gene was used for the molecular detection of strains using primers pair, βt2a (forward), 5′ GGTAACCAAATCGGTGCTGCTTTC 3′ and βt2b (reverse) 5′ ACCCTCAGTGTAGTGACCCTTGGC 3′ (31). PCR products subjected for sequence analysis and then sequences were manually verified by MEGA6 software package (https://www.megasoftware.net/) and aligned using the CLUSTALW algorithm. All sequences were compared to reference sequences in the Gen-Bank (NCBI) and CBS database via the nucleotide BLAST™ algorithm to obtain a definitive identification (similarity values ≥ 99%). Finally, all nucleotide sequences representative were deposited in the Gen-Bank database.

### Antifungal susceptibility assay.

Twofold serial dilutions of antifungals including, luliconazole (APIChem Technology, China) (from 0.00012 to 0.25 μg/mL), amphotericin B (Sigma - Aldrich, Germany) (from 0.125 to 16 μg/mL), voriconazole (Sigma- Aldrich, Germany) (from 0.0078 to 4 μg/mL), posaconazole (Sigma - Aldrich, Germany) (from 0.0312 to 4 μg/mL), and caspofungin (Sigma - Aldrich, Germany) (from 0.0078 to 1 μg/mL) were prepared in RPMI 1640 (Bio Idea, Iran). Antifungal susceptibility test was performed according to CLSI M38 A2 (32). A standard suspension (0.5 McFarland) of 48–72 hours cultures on SDA was prepared in sterile saline (0.85%) with 0.2% Tween 20 (Merck, Germany). Then, 100 μL of diluted suspension (1:50) and 100 μL of serial dilutions of each antifungal were added to each well of 96-well microplates. Micro-plates incubated at 35ºC for 24–72 hours and results were recorded as MIC or minimum effective concentration (MEC). Finally, MIC or MEC range, MIC_50_ or MEC_50_, MIC_90_ or MEC_90_ and MIC geometric (GM) or MECGM were calculated. CLSI or EUCAST have not been defined any clinical or epidemiologic breakpoints/cut-offs for amphotericin B, voriconazole, posaconazole, caspofungin and *Aspergillus* species. Strains susceptibility or resistance to each antifungals was evaluated according to commonly utilized breakpoints (Table 1) (33–38).

**Table 1.** Defined breakpoints of amphotericin B, voriconazole, posaconazole and caspofungin for *Aspergillus niger* sensu lato

**Antifungals**	**MIC or MEC (μg/mL)**	

	**Sensitive**	**Resistance**
Amphotericin B	≤2	>2
Posaconazole	≤0.5	>0.5
Voriconazole	≤1	>1
Caspofungin	≤0.06	>0.06
Luliconazole	Undefined	Undefined

MIC, Minimum inhibitory concentration; MEC, Minimum effective concentration

### Statistical analysis.

The Chi-squared test using the Social Science Statistics software (Online) was applied to determine the significant between variables and P value < 0.05 is considered as significance level.

## RESULTS

### Molecular detection of isolates.

37 clinical isolates of black *Aspergillus* were detected using molecular and sequencing techniques. *Aspergillus niger* (21, 56.8%) was the common strain followed by, *A. tubingensis* (11, 29.8%), *A. luchuensis* (1, 2.7%), and black *Aspergillus* strains (4, 10.8%) (Table 2). Furthermore, out of 30 environmental black *Aspergillus* isolates, 15 (50%) was identified as *A. niger* followed by, *A. tubingensis* (13, 43.3%), *A. piperis* (1, 3.3%) and black *Aspergillus* strains (1, 3.3%). However, we could not identified four clinical and one environmental black *Aspergillus* strains, using molecular technique due to inadequate DNA sample size.

**Table 2.** Clinical and environmental black *Aspergillus* strains

**Sources**	**Morphological identification**	**Molecular identification**
Clinical isolates (37 isolates)	*Aspergillus niger*	*A. niger*, sensu stricto (21)
	sensu lato	*A. tubingensis* (11)
		*A. luchuensis* (1)
	Black *Aspergillus* strains (4)	************

Environmental isolates (30 isolates)	*Aspergillus niger*	*A. niger*, sensu stricto (15)
	sensu lato	*A. tubingensis* (13)
		*A. piperis* (1)
	Black *Aspergillus* strains (1)	************

### Clinical isolates.

The lowest MIC range (0.00024–0.125 μg/mL), MIC_50_ (0.00195 μg/mL), MIC_90_, (0.125 μg/mL) and MICGM (0.00295 μg/mL) was attributed to luliconazole (Table 3). The MEC range for all clinical *Aspergillus* species was 0.0078–1 μg/ml for caspofungin. In addition, the 50% and 90% MEC (MEC_50_, MEC_90_) values were 0.125 and 0.5 μg/ml for caspofungin, respectively. Totally, the 54.1% of isolates were resistant to caspofungin. The results have shown that the MIC range of amphotericin B for tested isolates was 0.25–16 μg/mL. However, MIC_50_, MIC_90_ was similar, 8 μg/mL. The highest resistant rate (86.5%) was found for amphotericin B. The MIC ranges for clinical isolates of black *Aspergillus* strains were 0.0078–4 and 0.0625–4 μg/mL of voriconazole and posaconazole, respectively. However, the MICGM for voriconazole (0.77 μg/mL) was lower than posaconazole (1.45 μg/mL). In our study, 29.7% and 83.8% of isolates were resistant to voriconazole and posaconazole, respectively.

**Table 3.** The antifungal susceptibility pattern of 67 (37 clinical and 30 environmental) strains of black *Aspergillus*

**Clinical isolates of *Aspergillus* (37 isolates)**						
**Luliconazole**	**N**	**MIC range (μg/mL)**	**MIC_50_ (μg/mL)**	**MIC_90_ (μg/mL)**	**MICGM (μg/mL)**	**R (%)**
*Aspergillus niger*	21	0.00024 – 0.125	0.00195	0.125	0.00378	-
*A. tubingensis*	11	0.00024 – 0.125	0.00195	0.00391	0.00251	-
*A. luchuensis*	1	0.00098	-	-	-	-
Black *Aspergillus*	4	0.00049 – 0.00391	-	-	-	-
Total	37	0.00024 – 0.125	0.00195	0.125	0.00295	-

**Amphotericin B**	**N**	**MIC range (μg/mL)**	**MIC_50_ (μg/mL)**	**MIC_90_ (μg/mL)**	**MICGM (μg/mL)**	**R (%)**
*A. niger*	21	0.25 – 8	8	8	4.56	17 (81%)
*A. tubingensis*	11	4 – 16	8	8	8	11 (100%)
*A. luchuensis*	1	1	-	-	-	-
Black *Aspergillus*	4	4 – 8	-	-	-	4 (100%)
Total	37	0.25 – 16	8	8	5	32 (86.5%)

**Voriconazole**	**N**	**MIC range (μg/mL)**	**MIC_50_ (μg/mL)**	**MIC_90_ (μg/mL)**	**MICGM (μg/mL)**	**R (%)**
*A. niger*	21	0.0625 – 2	1	2	0.99	5 (23.8%)
*A. tubingensis*	11	0.5 – 4	1	2	1.20	4 (36.4%)
*A. luchuensis*	1	0.0078	-	-	-	-
Black *Aspergillus*	4	0.5 – 2	-	-	-	2 (50%)
Total	37	0.0078 – 4	1	2	0.77	11 (29.7%)

**Posaconazole**	**N**	**MIC range (μg/mL)**	**MIC_50_ (μg/mL)**	**MIC_90_ (μg/mL)**	**MICGM (μg/mL)**	**R (%)**
*A. niger*	21	0.0625 –4	2	2	1.26	17 (81%)
*A. tubingensis*	11	0.125 – 4	2	4	2.13	10 (90.9%)
*A. luchuensis*	1	0.5	-	-	-	-
Black *Aspergillus*	4	0.25 – 4	-	-	-	4 (100%)
Total	37	0.0625 – 4	2	4	1.45	31 (83.8%)

**Caspofungin**	**N**	**MEC range (μg/mL)**	**MEC_50_ (μg/mL)**	**MEC_90_ (μg/mL)**	**MECGM (μg/mL)**	**R (%)**
*A. niger*	21	0.0078 – 1	0.125	0.5	0.099	11 (52.4%)
*A. tubingensis*	11	0.032 – 0.5	0.125	0.5	0.133	7 (63.6%)
*A. luchuensis*	1	0.032	-	-	-	-
Black *Aspergillus*	4	0.0625 – 0.25	-	-	-	2 (50%)
Total	37	0.0078 – 1	0.125	0.5	0.107	20 (54.1%)

**Environmental isolates of *Aspergillus* (30 isolates)**						

**Luliconazole**	**N**	**MIC range (μg/mL)**	**MIC_50_ (μg/mL)**	**MIC_90_ (μg/mL)**	**MICGM (μg/mL)**	**R (%)**
*Aspergillus niger*	15	0.00098 – 0.0078	0.00195	0.00391	0.00214	-
*A. tubingensis*	13	0.00049 – 0.00781	0.00195	0.00391	0.00195	-
*A. piperis*	1	0.00195	-	-	-	-
Black *Aspergillus*	1	0.00049	-	-	-	-
Total	30	0.00049 – 0.00781	0.00195	0.00391	0.00195	-

**Amphotericin B**	**N**	**MIC range (μg/mL)**	**MIC_50_ (μg/mL)**	**MIC_90_ (μg/mL)**	**MICGM (μg/mL)**	**R (%)**
*A. niger*	15	2 – 16	8	16	6.964	14 (93%)
*A. tubingensis*	13	4 – 8	4	8	5.508	13 (100%)
*A. piperis*	1	4	-	-	-	-
Black *Aspergillus*	1	4	-	-	-	-
Total	30	2 – 16	8	8	6.063	29 (96.7%)

**Voriconazole**	**N**	**MIC range (μg/mL)**	**MIC_50_ (μg/mL)**	**MIC_90_ (μg/mL)**	**MICGM (μg/mL)**	**R (%)**
*A. niger*	15	0.125 – 2	1	2	0.6300	2 (13.3%)
*A. tubingensis*	13	0.0625 – 2	0.5	2	0.4261	2 (15.4%)
*A. piperis*	1	0.125	-	-	-	-
Black *Aspergillus*	1	0.0625	-	-	-	-
Total	30	0.0625 – 2	0.5	2	0.4665	4 (13.3%)

**Posaconazole**	**N**	**MIC range (μg/mL)**	**MIC_50_ (μg/mL)**	**MIC_90_ (μg/mL)**	**MICGM (μg/mL)**	**R (%)**
*A. niger*	15	0.5 –4	2	4	1.8234	14 (93%)
*A. tubingensis*	13	0.125 – 4	2	4	1.1125	7 (53.8%)
*A. piperis*	1	0.5	-	-	-	-
Black *Aspergillus*	1	0.0625	-	-	-	-
Total	30	0.0625 – 4	2	4	1.2599	21 (70%)

**Caspofungin**	**N**	**MEC range (μg/mL)**	**MEC_50_ (μg/mL)**	**MEC_90_ (μg/mL)**	**MECGM (μg/mL)**	**R (%)**
*A. niger*	15	0.0078 – 0.25	0.032	0.25	0.0412	3 (20%)
*A. tubingensis*	13	0.0078 – 0.5	0.0625	0.5	0.0733	6 (46.2%)
*A. piperis*	1	0.0625	-	-	-	-
Black *Aspergillus*	1	0.0078	-	-	-	-
Total	30	0.0078 – 0.5	0.0625	0.25	0.0507	9 (30%)

N, number; MEC, Minimum effective concentration; MIC, Minimum inhibitory concentration; GM, Geometric; R, Resistant

### Environmental isolates.

The results summarized in Table 3 show the *in vitro* susceptibilities of 30 environmental *Aspergillus* Nigri against several antifungals. The same as clinical isolates, the lowest MIC range was 0.00049–0.00781 μg/ml for luliconazole. Moreover, the MIC_50_, MIC_90_ and MICGM of luliconazole were 0.00195, 0.00391 and 0.00195 μg/ml, respectively. The MEC range, MEC_50_, MEC_90_ and MECGM for caspofungin were 0.0078-0.5, 0.0625, 0.25, and 0.0507 μg/ml, respectively. Furthermore, 30% of environmental strains were resistant to caspofungin. As shown in Table 3, the MIC range for amphotericin B was 2–16 μg/ml followed by, MIC_50_, MIC_90_ and MICGM were 8, 8 and 6.063 μg/ml, respectively. Moreover, 96.7% of strains were resistant to amphotericin B. Totally, the MIC range voriconazole for environmental isolates of *Aspergillus* was 0.0625–2 μg/ml, whereas MIC_90_ 2 μg/ml, MIC_50_ 0.5 and MICGM 0.4665 μg/ml). Our results indicated that only 4 (13.3%) strains were resistant to voriconazole. The tested isolates were inhibited at MIC range 0.0625–4 μg/ml by posaconazole. Furthermore, the MIC_50_, MIC_90_ and MICGM were 2, 4 and 1.2599 μg/ml, respectively. In addition, 70% of strains were resistant to posaconazole.

Caspofungin was significantly more effective against environmental than clinical strains (P = 0.048) of black *Aspergillus* strains. However, the inhibitory effect of amphotericin B, posaconazole and voriconazole was similar against both tested strains (clinical and environmental) (amphotericin B, P=0.147; voriconazole, P=0.109; posaconazole, P=0.178). When we compared the effect antifungals against *A. niger* and *A. tubingensis* strains, it found that caspofungin was more effective on *A. niger* with environmental sources than clinical strains (P=0.0482). Whereas, the effect of other antifungals against both species was not significant.

Our results showed that 32 (86.5%) of clinical strains were resistant to 2, 3 or 4 antifungals, 2 (5.4%) isolates were resistant to one antifungal and 3 (8.1%) isolates were fully susceptible to all antifungals (Table 4). Two strains of *A. tubingensis*, one *A. niger* and one black *Aspergillus* strains were resistant to all antifungals (except luliconazole). On the other hand, 21 (70%) of environmental strains were resistance to 2 –4 antifungals and only 30% of strains were resistance to one antifungals (Table 5). Two strains of *A. niger* and one *A. tubingensis* were resistant to all antifungals (except luliconazole).

**Table 4.** Drug resistance against tested antifungals among 37 clinical strains

**Clinical strains**	**Accessions numbers**	**Antifungal drugs**				

		**LUL**	**POS**	**VOR**	**AMP**	**CAS**
*Aspergillus niger*	LC441157	0.125	R	S	R	R
*A. niger*	LC456335	0.125	S	S	R	R
*A. niger*	LC456339	0.125	S	S	R	R
*A. niger*	LC441167	0.125	R	S	R	R
*A. tubingensis*	LC456340	0.125	R	S	R	R
*A. niger*	LC456341	0.01561	R	S	R	R
*A. niger*	LC441156	0.00781	R	S	R	R
*A. niger*	LC456337	0.00781	R	S	S	S
*A. tubingensis*	LC456338	0.00391	R	R	R	R
Black *Aspergillus*	********	0.00391	R	S	R	S
*A. tubingensis*	LC441168	0.00391	R	R	R	R
*A. niger*	LC441162	0.00391	R	S	R	R
*A. niger*	LC456326	0.00195	R	R	R	S
*A. tubingensis*	LC456298	0.00195	R	R	R	S
*A. tubingensis*	LC456302	0.00195	R	R	R	S
Black *Aspergillus*	********	0.00195	R	R	R	S
*A. tubingensis*	LC456301	0.00195	R	S	R	R
*A. niger*	LC441161	0.00195	R	S	R	R
*A. tubingensis*	LC441169	0.00195	R	S	R	R
*A. niger*	LC441158	0.00195	R	R	R	S
*A. tubingensis*	LC456303	0.00195	R	S	R	S
*A. niger*	LC456323	0.00195	R	R	R	S
Black *Aspergillus*	********	0.00195	R	R	R	R
*A. niger*	LC456336	0.00195	R	S	R	S
*A. tubingensis*	LC441171	0.00195	S	S	R	R
*A. niger*	LC441163	0.00098	R	S	R	S
*A. niger*	LC441159	0.00098	R	S	R	R
*A. niger*	LC441160	0.00098	R	S	S	S
*A. niger*	LC441165	0.00098	R	R	R	R
*A. tubingensis*	LC441170	0.00098	R	S	R	S
*A. niger*	LC441164	0.00098	R	R	R	S
*A. niger*	LC456320	0.00098	R	S	R	R
*A. luchuensis*	LC456304	0.00098	S	S	S	S
Black *Aspergillus*	********	0.00049	S	S	R	R
*A. tubingensis*	LC456297	0.00024	R	S	R	R
*A. niger*	LC441166	0.00024	S	S	S	S
*A. niger*	LC441155	0.00024	S	S	S	S

LUL, Luliconazole; POS, Posaconazole; VOR, Voriconazole; AMP, Amphotericin B; CAS, Caspofungin; R, Resistance: S, Susceptible

**Table 5.** Drug resistance against tested antifungals among 30 environmental strains

**Environmental strains**	**Accessions number**	**Antifungal drugs**				

		**LUL**	**POS**	**vOR**	**AMP**	**CAS**
*Aspergillus niger*	LC456329	0.00781	R	S	R	S
*A. tubingensis*	LC456309	0.00781	R	R	R	S
*A. niger*	LC456331	0.00391	R	S	R	S
*A. niger*	LC456322	0.00391	R	S	R	S
*A. niger*	LC456334	0.00391	R	R	R	R
*A. tubingensis*	LC456316	0.00391	R	R	R	R
*A. niger*	LC456318	0.00391	R	S	R	S
*A. niger*	LC456324	0.00195	R	S	R	S
*A. tubingensis*	LC456315	0.00195	R	S	R	R
*A. niger*	LC456332	0.00195	R	S	R	S
*A. tubingensis*	LC456307	0.00195	S	S	R	S
*A. niger*	LC456325	0.00195	R	S	R	S
*A. niger*	LC456327	0.00195	R	S	S	S
*A. tubingensis*	LC456311	0.00195	S	S	R	S
*A. niger*	LC456328	0.00195	R	S	R	R
*A. tubingensis*	LC456312	0.00195	S	S	R	S
*A. tubingensis*	LC456306	0.00195	R	S	R	R
*A. tubingensis*	LC456314	0.00195	R	S	R	R
*A. tubingensis*	LC456300	0.00195	R	S	R	S
*A. tubingensis*	LC456308	0.00195	R	S	R	R
*A. niger*	LC456330	0.00195	R	S	R	S
*A. tubingensis*	LC456299	0.00195	S	S	R	S
*A. piperis*	LC456305	0.00195	S	S	R	S
*A. niger*	LC456321	0.00098	S	S	R	S
*A. niger*	LC456333	0.00098	R	S	R	S
*A. tubingensis*	LC456313	0.00098	S	S	R	S
*A. niger*	LC456317	0.00098	R	R	R	R
*A. niger*	LC456319	0.00098	R	S	R	S
Black *Aspergillus*	********	0.00049	S	S	R	S
*A. tubingensis*	LC456310	0.00049	S	S	R	R

LUL, Luliconazole; POS, Posaconazole; VOR, Voriconazole; AMP, Amphotericin B; CAS, Caspofungin; R, Resistance: S, Susceptible

## DISCUSSION

*Aspergillus* strains isolated from clinical and air borne samples were identified using classical morphological features and molecular methods. In the present study, *A. tubingensis, A. luchuensis* and *A. piperis* were identified as the cryptic species of *A. niger* sensu lato by the sequence analysis of β-tubulin gene. Several reports have shown that *A. niger* is generally as common causative agent of otomycosis and one of the most important agent for invasive aspergillosis (20, 22, 26, 39–41). However, this species cannot be reliably detected from other cryptic members of *Aspergillus* section Nigri using conventional morphological methods. Molecular tools with sequence-based techniques such as partial sequence of the β-tubulin gene are presented as the most valuable method for *A. niger* Nigri species assignment (4, 21). These molecular techniques are indicating that this species comprises 19 cryptic species (4, 16, 21) with more prevalence of *A. niger* sensu stricto and *A. tubingensis* (16, 42).

Our results showed that, although the luliconazole MIC ranges for strains were extremely low, this range for environmental strains (0.00781–0.00049 μg/ml) was lower than clinical strains (0.125 – 0.00024 μg/ml). As shown in Table 5, only five clinical strains (*A. niger* sensu stricto, 4 isolates and *A. tubingensis*, 1 isolate) have a MIC = 0.125 μg/ml. 30/30 (100%) of environmental and 83.8% of clinical strains had the lowest MICs (MICs < 0.00781 μg/ml) against luliconazole. Moreover, the MICGM for environmental and clinical strains were 0.00195 and 0.00295 μg/ml, respectively. Some studies have shown a high efficacy of luliconazole against dermatophytes and onychomycosis agents both *in vivo* and *in vitro* (1, 2, 7, 8, 43). Furthermore, recently a few studies examined the potency of luliconazole against different species of *Candida, A. fumigatus, A. terreus* and *Fusarium* species (5, 6, 44, 45). However, the potency profile of luliconazole against *A. niger* complex is unknown. Abastabar et al. (3) and Omran et al. (6) were tested luliconazole against *A. fumigatus* and *A. flavus,* and found that the antifungal has the lowest MICs against *A. fumigatus* (MIC90 0.002 μg/ml) and *A. flavus* (MIC_90_ 0.032 μg/ml), respectively.

There are the limited data in *in vitro* efficacy of caspofungin against black *Aspergillus* strains from clinical and environmental sources. While, the clinical and environmental strains had the same MIC ranges for caspofungin, the resistant to antifungal showed the clear differences between clinical and environmental strains (P = 0.048), where the clinical isolates showed higher resistant rate than the environmental strains. In a report by Badali et al. only 6.1% of environmental strains of *A. niger* were resistant to caspofungin and all clinical isolates ranged at 0.008 – 0.063 μg/ml (21). In agree with our study Araujoa et al., revealed significantly higher MIC values to caspofungin in the case of non-*fumigatus* clinical than environmental strains (46).

The *in vitro* activities of posaconazole, voriconazole, and amphotericin B against clinical *Aspergillus* strains have been reported by Arikan et al. (10). They reported that voriconazole was the most active anti-fungal against *A. niger*. Comparable to our results, voriconazole was more potent than the other tested antifungals (with exception luliconazole) against both clinical and environmental strains. Similar to our study, Hashimoto et al., showed no remarkable differences between the MIC distribution rate of voriconazole against clinical and environmental isolates (15). Furthermore, all tested *A. niger* (environment and clinical isolates) were susceptible to both amphotericin B and voriconazole in Misra et al., research (47). *Aspergillus tubingensis* resistant strains to amphotericin B was very common both in environment and clinical settings, followed by posaconazole, caspofungin, and voriconazole. However, the resistant rate to amphotericin B was lower among environmental than clinical strains. Hashimoto et al. finding suggests that *A. tubingensis* is intrinsically resistant to azole antifungals (15). Antifungal susceptibility testing of our *A. tubingensis* strains revealed 90.9% and 53.8% of clinical and environmental isolates were resistant to posaconazole.

## CONCLUSION

In conclusion, luliconazole versus amphotericin B, voriconazole, posaconazole and caspofungin is a potent antifungal for *Aspergillus* Nigri complex. The *in vitro* extremely antifungal efficacy against black *Aspergillus* strains of luliconazole, is different from those of other used antifungals. The MIC range, MIC_50_, MIC_90_ and MICGM of luliconazole against black *Aspergillus* strains were the lowest among the representative tested antifungals. These results suggest luliconazole can be a viable option for the treatment of infections due to black *Aspergillus* strains and should be further investigated *in vivo*.
